# About 3D Incompressible Flow Reconstruction from 2D Flow Field Measurements

**DOI:** 10.3390/s22030958

**Published:** 2022-01-26

**Authors:** Laura Fabbiano, Paolo Oresta, Aimé Lay-Ekuakille, Gaetano Vacca

**Affiliations:** 1 Department of Mechanics, Mathematics and Management, Polytechnic University of Bari, Via E. Orabona, 4, 70125 Bari, Italy; paolo.oresta@poliba.it (P.O.); gaetano.vacca@poliba.it (G.V.); 2 Department of Innovation Engineering, University of Salento, Via per, Monteroni, 73100 Lecce, Italy; aime.lay.ekuakille@unisalento.it

**Keywords:** fluid dynamics measurements, uncertainty evaluation, hybrid procedure, 3D flow velocity reconstruction, sensor signal processing, machine learning, Kriging, Sobol sensitivity

## Abstract

In this paper, an assessment of the uncertainty affecting a hybrid procedure (experimental/numerical) is carried out to validate it for industrial applications, at the least. The procedure in question serves to depict 3D incompressible flow fields by using 2D measurements of it and computing the third velocity component by means of the continuity equation. A quasi-3D test case of an incompressible flow has been inspected in the wake of a NACA 0012 airfoil immersed in a forced flow of water running in a rectangular open channel. Specifically, starting from a 2D measurement data in planes orthogonal to the stream-wise direction, the computational approach can predict the third flow velocity component. A 3D ADV instrument has been utilized to measure the flow field, but only two velocity components have been considered as measured quantities, while the third one has been considered as reference with which to compare the computed component from the continuity equation to check the accuracy and validity of the hybrid procedure. At this aim, the uncertainties of the quantities have been evaluated, according to the GUM, to assess the agreement between experiments and predictions, in addition to other metrics. This aspect of uncertainty is not a technical sophistication but a substantial way to bring to the use of a 1D and 2D measurement system in lieu of a 3D one, which is costly in terms of maintenance, calibration, and economic issues. Moreover, the magnitude of the most relevant flow indicators by means of experimental data and predictions have been estimated and compared, for further confirmation by means of a supervised learning classification. Further, the sensed data have been processed, by means of a machine learning algorithm, to express them in a 3D way along with accuracy and epoch metrics. Two additional metrics have been included in the effort to show paramount interest, which are a geostatistical estimator and Sobol sensitivity. The statements of this paper can be used to design and test several devices for industrial purposes more easily.

## 1. Introduction

The characterization of the coherent structures populating the flow domain (such as the trailing wake behind bluff body, low-speed streaks in channel flows, large scale circulation in bounded heat transfer flows, convective boundary layer), is crucial in theoretical and applied fluid dynamics. In the literature, the coherent structures have been depicted by means of vorticity and stress tensors that are based on the spatial distribution of the flow velocity. The velocity field evaluation shows a crucial relevance for the design of devices in engineering applications [1]. Among others, the flow field surrounding an airfoil is appealing for the improvement in aerospace, automotive, marine challenging applications, as well as for wind turbines design and/or operation. Its evaluation has always required many efforts to be accurately measured or computed.

In the last decade, algorithms and data logging capabilities have strongly increased the accuracy and the user-friendly management of the experimental facilities and computational techniques. For instance, high resolution measurements in space and in time about the three components of the velocity of turbulent flows have been obtained by means of 3D laser or acoustic instrumentation, as well as by DNS or LES numerical techniques from a computational point of view. If such experimental technologies are still expensive, the cited numerical techniques are, instead, time consuming and require massive computational equipment. In both cases, the worse drawback consists of the difficulty to have their availability in real industrial framework, where more traditional instrumentation is used for experimental acquisitions (hot wire, Pitot tube, cylindrical probe, and similar), or commercial software and equipment for computations.

The aim of this paper is to show that it is possible to numerically reconstruct a real 3D flow field without losing significant accuracy in industrial applications, by means of simpler plane acquisitions through 2D experimental equipment, thus reducing the costs of the equipment for the experimental activities, with the subsequent lowering of the calibration and maintenance problems.

In this work, the 3D ADV (acoustic Doppler velocimetry) experimental technology has been utilized to validate the approach, because the only accurate instrumentation available is intrusive as well as expensive [2]. Although this probe might be taken into consideration for measuring three-dimensional flow velocities in both laboratory and field applications [3,4,5,6], for which other more performing 3D measurement techniques such as laser Doppler anemometers or PIV are much less common due to their cost, it suffers from some typical error sources in measurements such as: Doppler ambiguity, spatial averaging, mean flow shear, phase distortion, sampling errors, and air concentration [7,8,9], besides the insertion error. In order to deal with these biases in the measurements of the velocity components, the density of particles seeded into the flow and other control parameters should be set preliminarily via a calibration procedure [10], which has been opportunely set here before the campaign of acquisitions. 

The authors here intend to validate the hybrid approach for reconstructing a 3D flow field from 2D measurement acquisitions and the solution of the local continuity equation [11] to determine the third velocity component by assessing the efficiency of the procedure by the accuracy estimation. The flow field velocity acquisitions have been made by means of the 3D instrumentation, taking only two of them as measured and using the third one as a reference value for evaluating the goodness of the numerical approach. Specifically, we have made the velocity measurements in an open channel, where an airfoil was already immersed for other aims, utilizing its wake region as a work area. The third velocity component, evaluated by the dedicated algorithm, has been compared with the experimental value given by the instrument itself. The uncertainty analysis has been carried out in order to assess the accuracy of the hybrid procedure.

This paper is further organized into seven sections, besides the Introduction: Section 2 is devoted to the experimental procedure and presents results useful to demonstrating the validity of the mixed procedure by means of the uncertainty analysis, thus pondering the accuracy of the methodology.

In the Section 3, we describe the algorithm and the numerical analysis. From the finite difference discretization of the continuity equation, the value of the stream-wise velocity component is computed as a function of the measured values of the other two. Further, in Section 4, the results from the experiment and computations are presented and compared, while Section 5 treats the uncertainty evaluations for the computed quantities. Section 6 deals with the use of dedicated metrics and clustering for better expressing the results under another lens, in particular, with machine learning, in an effort to carry out a clustering of data components related to the searched third axis. This clustering is important, being able to make the relationship coherent among the recovered data. For further understanding, we have promoted, as illustrated in Section 7, the use of geostatistics (variogram, and Kriging estimator), and the Sobol-based sensitivity analysis. It is the first to use both indicators regarding the issues of this topic. The last section is devoted to the discussion about some evaluable parameters from the computations performed to better characterize the flow under the study and conclusions derived.

The numerical analysis is accurate to the first order for simplicity of computations since the artificial fluctuations in the velocity field are smoothed. The method can be easily developed with a higher order discretization scheme (by using more planes of measurement with finer spacing) without changing the main core of the procedure, in order to obtain better accuracy.

## 2. Experimental Setup and Procedure

**Figure 1:**
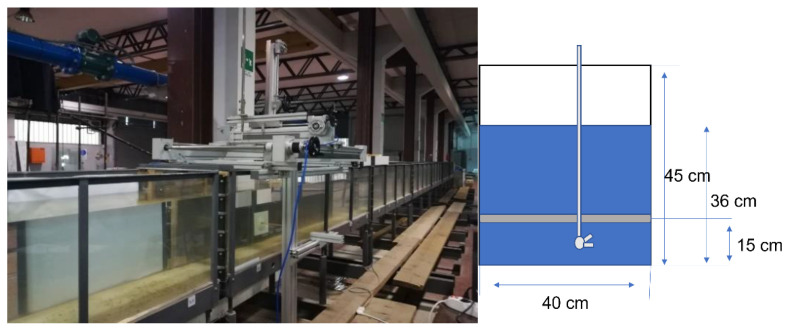
Water channel for experiments (**left**) and its cross-section (**right**).

The measurements are carried out in a channel with a rectangular section *h* = 45 cm heigh and w=40 cm wide (Figure 1). The wet area of the flow section is 36 cm high and 40 cm wide.

**Figure 2:**
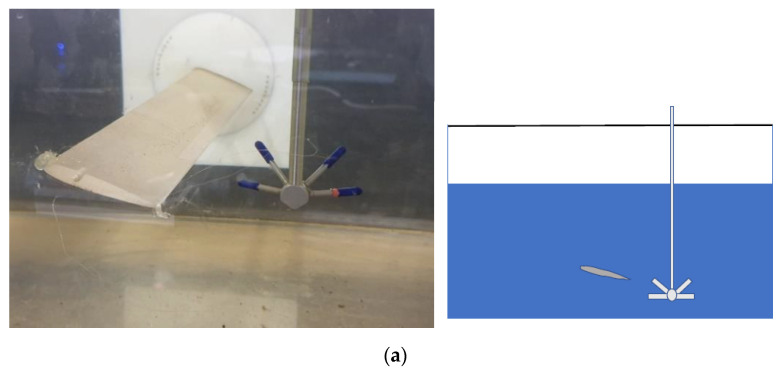

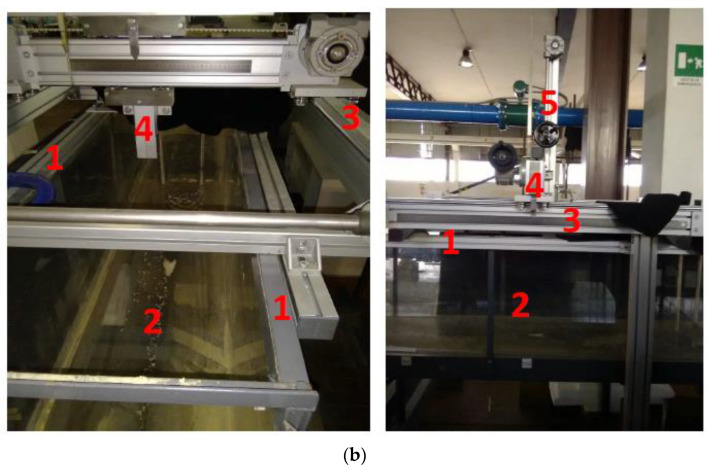
(**a**) NACA 0012 airfoil profile: acquisition probe Vectrino ADV 3D placed in the wake (**left**), and its cross-section (**right**). (**b**) Longitudinal view (**left**), and cross-view of the channel (**right**): channel chassis/skeleton keeping the glass side (1), channel inner part allowing the flow (2), 3D instrument rail (3), head bringing the 3D instrument directed to the channel for measurement (4), vertical rail allowing an up-and-down displacement of the 3D instrument (5).

In the volume domain of observation, an airfoil is placed at 15 cm from the bottom of the duct (see Figure 2a). Its chord is 5 cm long and it has 20° angle of attack in order to increase the turbulence in the wake downstream. Additional details of the channel are encompassed in Figure 2b, where one can see the main reasons of experimentally locating the 3D instrument. Certainly, it is difficult to populate an infrastructure based on a channel, especially for long distances, with a 3D instrument, depending on if we need sectional measurements. It could be appropriate to mount a 3D instrument at one section, even in a provisional way, and locate conventional instruments base on 1D/2D measurements in other points. 

The goal of the experiment is to acquire the velocity components of the flow in planes orthogonal to the stream-wise direction to obtain the stream-wise component *u* by using a numerical algorithm. The values of *u* computed in each cell point have been compared with the respective values measured by the ADV at the same point.

**Figure 3:**
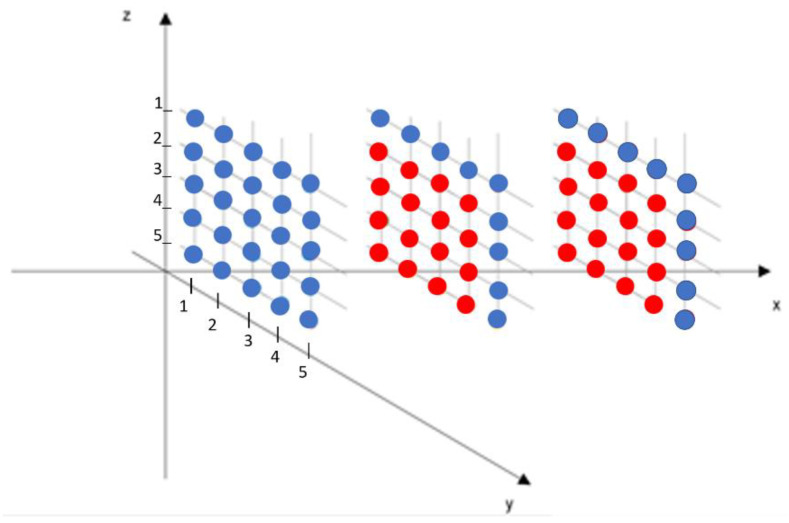
Subdivision of the fluid domain in 3 measuring grids of 25 points each with equal spacing steps Δ*x* = Δ*y* = Δ*z* = 5 cm. The colored blue points, different from the red ones, are considered boundary points for the numerical integration of the continuity equation.

The (*y*, *z*) planes with their 25 equally spaced measuring points, as depicted in Figure 3, are located at three different positions in *x*, and the total points in the volume investigated are equal to 75; starting at about 5 cm from the bottom of the channel, measurements are taken up to 30 cm high, where the velocity is considered to be free stream. The inflow plane of the considered control volume (here consisting of three planes only) is located 5 cm downstream the airfoil trailing edge. Only for the last two planes, the *u* components have been computed, as the inflow plane has been considered as a known boundary from the measurements.

The boundary conditions have been imposed at the inflow plane points and opportune contour points of the computational planes, where the velocity components were known from ADV measurements (see Figure 3) according to the discretization scheme adopted for the solution of the continuity equation.

The measured set of velocity values are sampled at each cell point of the chosen control volume at a fixed sampling frequency for a given time lapse and then averaged in time, according to the following relationship:

(1) $${\bar{u}}_{i,j,k}=\frac{1}{N_{sample}}t\;\overset{t_{end}}{\underset{t=0}{\sum }}u_{i,j,k}\left(t\right);\;{\bar{v}}_{i,j,k}=\frac{1}{N_{sample}}\;\overset{t_{end}}{\underset{t=0}{\sum }}v_{i,j,k}\left(t\right);{\bar{w}}_{i,j,k}=\frac{1}{N_{sample}}\;\overset{t_{end}}{\underset{t=0}{\sum }}w_{i,j,k}\left(t\right)$$

where $N_{sample}$ are the acquired values of each velocity component in the chosen recording time. As the acquisition of a signal, representing the time trend of a physical quantity can be designated as a random process, its stationarity is proved if the statistics computed for it are time invariant. A criterion to be satisfied to confirm such a property, justifying the use of the above relations for the time-average value evaluations, is given by the respect of the WWS (Wise Sense Stationary) condition [12,13], which imposes that:

- The mean value of the signal is time-invariant.
- The signal variance is time-invariant.
- The signal auto-correlation function depends only on the time-lag used for its computation.

All these positions have been verified for each acquired signal by replicating the acquisition three times at least.

The sampling frequency has been fixed equal to 0.1 kHz with a recording time of 60 s for each grid node.

**Figure 4:**
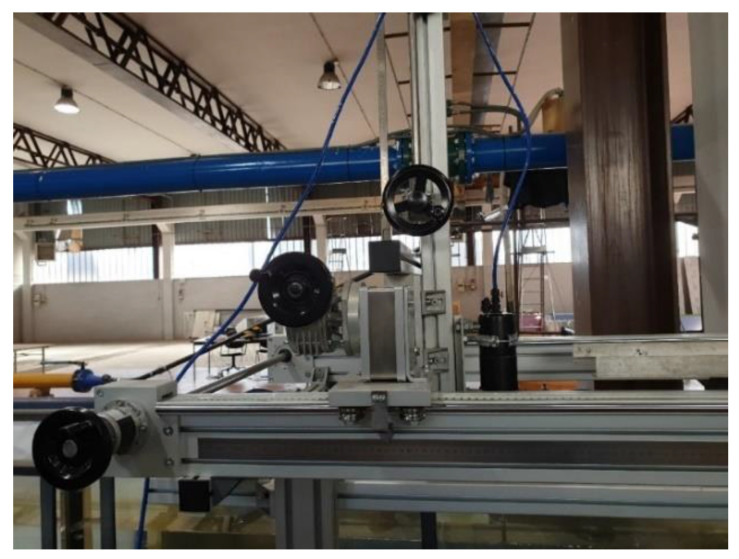
3D instrument moving system of graduation scales.

The instrumentation consists of the 3D Vectrino ADV (Figure 2), whose main characteristics have been summarized in Table 1, and a high precision 3D system of graduation scales to determine the locations of the measurement points, Figure 4.

## 3. Numerical Procedure

In the case of incompressible steady flow, the density does not change in time (as well as in space). The continuity equation in the Eulerian frame reads as follow:

(2) $$\rho \left(\nabla \cdot V\right)=\rho \left(\frac{\partial u}{\partial x}+\frac{\partial v}{\partial y}+\frac{\partial w}{\partial z}\right)=0.$$

The variation of the velocity components in the space (x, y, z) are coupled by the continuity equation. Thus, if we suppose that the derivatives of the two components (v, w) are computable from their measurements, we can estimate the third component (u) using the continuity equation in discrete form. We refer to backward (or forward) finite difference approaches for which the space derivative can be approximated at the first order as follows:

(3) $$f^{\prime \left(t_{n}\;\right)}=\frac{f\left(t_{n}\right)-f\left(t_{n-1}\;\right)}{\Delta s}\vee \;\;f^{\prime \left(t_{n}\;\right)}=\frac{f\left(t_{n+1}\right)-f\left(t_{n}\;\right)}{\Delta s},\;$$

where f is the function defined in $Ƒ\in C^{k}\;\left[\left(a,b\right)\right]$ that has been derived on a step Δs.

The numerical scheme is first order backward for the stream-wise component, while first order forward finite differences have been used for the other two derivative terms of the continuity equations, according to the numbering of the cell points of Figure 3, computed in the two planes along the *x* direction. The experimental data are indicated with the overbar, whereas the theoretical prediction is indicated without the overbar. The subscription (i,j,k) defines the indices in the discrete space corresponding to the continuous space (x,y,z). For all the grid points (i,j,k) of the computational domain, the theoretical velocity $u_{i,j,k}$, can be calculated by the average (in time) velocities $\bar{w}$ and $\bar{v}$, measured at the appropriate cell points. The value $u_{i,j,k}$ is then compared with the measured value ${\bar{u}}_{i,\;j,\;k}$.

The derivative of the velocities v and w are computed for all the point i,j,k using the first order finite difference discretization of the derivative terms as,

(4) $$\frac{\partial v_{i,j,k}\;}{\partial y}=\frac{{\bar{v}}_{i,j+1,k}-{\bar{v}}_{i,j,k}}{\Delta y};\;\frac{\partial w_{i,j,k}}{\partial z}=\frac{{\bar{w}}_{ij,k+1}-{\bar{w}}_{i,j,k}}{\Delta z}\;,$$

(depending on the number of the planes of measurement, higher orders of approximation can be used as well).

The value of the unknown component u can thus be computed from the continuity equation by expressing the first order finite difference of the relative derivative term ∂u/∂x evaluated in (i,j,k) generic point as, from Equation (2):

(5) $$u_{i,j,k}=u_{i-1,j,k}-\left(\frac{\partial \bar{v}}{\partial y}+\frac{\partial \bar{w}}{\partial z}\right)\Delta x,$$

where the derivative terms are computed according to Equation (4) from the experimental data of the *v* and *w* velocity components respectively.

**Figure 5:**
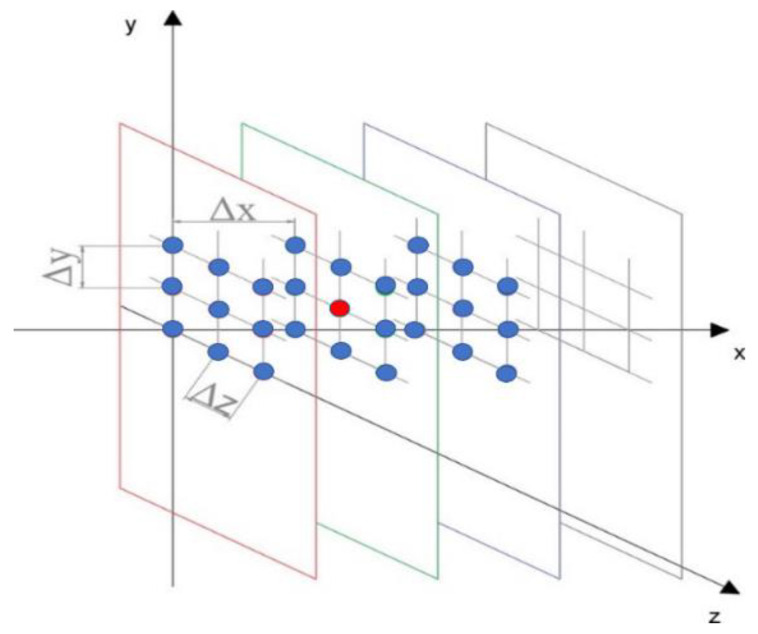
As an example in the central plane, the red cell means that the *u* component is computed from the continuity equation in function of the *v* and *w* components measured in the necessary grid points of the plane, according to the numerical scheme adopted.

The space control volume is discretized by a three-dimensional grid, where the cell points are spaced by equal displacements Δx, Δy and Δz in the three orthogonal directions, as indicated in Figure 5, and they coincide with the measuring points of the experimental procedure.

## 4. Experimental Results

In this section, we report the average (in time) values of the velocities measured by the campaign carried out in the cell points of the whole domain. The mean velocity in each point is obtained after despiking the acquired signals, processed on the MATLAB platform.

We define $U_{1},\;V_{1},\;W_{1}$ as the matrix of the average velocity components located at the grid points P(x,y,z) in the inflow plane 1, similarly, $U_{2},\;V_{2},\;W_{2}$ at the midplane and $U_{3},\;V_{3},\;W_{3}$ at the outflow plane of the experimented control volume. 

Their evaluations, from the acquired data, are summarized in Table 2, according to the row and column numberings of Figure 5.

As an example of results, the predicted *u* velocities at the middle plane are shown in the Table 3.

The last row in Table 3, corresponding to the height of 5 cm from the bottom, shows the stream-wise velocity components of the same order of magnitude of the other higher lines, although less precise, because those points are out of the boundary layer, but greatly influenced by this as well as by the noise on the *v* values, close to zero. 

Defining the percentage error as $\left(u_{2-calc}-u_{2-meas}\right)/u_{2-meas}$ the following errors Table 4 is obtained.

## 5. Uncertainty Evaluation

The uncertainty evaluation of the computed mean velocity $u_{2}$ is governed by the propagation law applied to Equation (5) [15]. 

The velocity can be estimated plugging in the previous equation the finite differences of the spatial derivatives of the velocities $v_{2}$ and $w_{2}$ as:

(6) $$u_{i,j,k}=u_{i,-1j,k}-\left(\frac{\left({\bar{v}}_{i,j+1,k}-{\bar{v}}_{i,j,k}\right)}{\Delta y}+\frac{\left({\bar{w}}_{i,j,k+1}-{\bar{w}}_{i,j,k}\right)}{\Delta z}\right)\Delta x,\;$$

with i=2, j=1.4 and k=2.5.

The contributions to the uncertainty of *u*, *v* and *w* − components depend on the repeatability errors (deriving from the acquisitions) and accuracy and spatial resolution of the instrumentation (type B uncertainty evaluation by rectangular distribution). In the proposed analysis, we neglect the resolution of the graduation scales and the bias due to the algorithm used, because the first is less significant with respect to the other contributions and the second, here a 1% order of magnitude, and minifying it through higher order numerical discretization.

As an example, we report, in Table 5, the matrix of the *u* mean square errors relative to the measures at the inflow control plane along *z*-direction, $S_{u_{1}}$.

Considering the Vectrino accuracy of $\pm \left(0.5\%\cdot \bar{v}\pm 1\right)\;\mathrm{mm}/s$, the accuracy values of the $u_{2-calc}$ from Equation (5) are reported in Table 6.

## 6. Machine Learning-Based Metrics

The machine learning approach, connected to classification learner, is based on a decision tree involving all quantities encompassed in Table 2. The classification techniques, within the artificial intelligence methods, are generally the following: (i) decision trees, here used; (ii) neural networks, a subset of machine learning; (iii) k-NN, k-Nearest Neighbor, and (iv) Genetic Algorithms [16]. 

**Figure 6:**
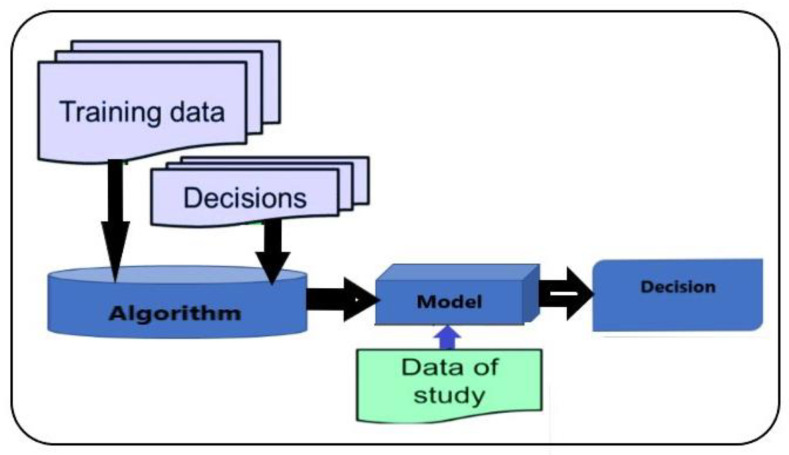
Proposed supervised learning classification algorithm.

In these tree structures, as per the flowchart reported in Figure 6, the sheets represent the values of the target-variable, and the branches correspond to combinations of input variables that lead to these values. In decision analysis, a decision tree can be used to explicitly represent the decisions made and the processes that lead to them. In learning and in data mining, a decision tree describes the data but not the decisions themselves, and the tree would be used as a starting point for the decision process [17].

The purpose of classification trees is to predict or explain the responses of a categorical dependent variable, which is why the techniques in this module are quite similar to the more traditional methods of Discriminant Analysis, Classification, Non-Parametric Tests and of Non-Linear Estimation. The flexibility of classification trees makes them an attractive analytical option, but one that should not completely replace more traditional methods. The training data are flow velocities along the three directions, *x*, *y*, *z*, that is

(7) $$\left(U_{1},U_{2},U_{3})=(u_{11},u_{12},\ldots u_{1n};u_{21},u_{22}\ldots u_{2n};u_{31},u_{32},\ldots u_{3n}\right)$$

where *U*_1_, *U*_2_, and *U*_3_ are the velocities along the three axes as reported in Table 2. 

The adopted classification is based on the decision due to the resubstituting estimator [18], with one of the classifiers along with the test sample estimator and the V-fold. It represents the proportion of observations misclassified by the classification model constructed from the entire sample. It is calculated as follows:

(8) $$Rd=\frac{1}{N}\sum_{i-1}^{N}X(d(x_{n})\neq j_{n})$$

where *U* represents the function of the indicator:
X=1, if the $X\left(d\left(x_{n}\right)\neq j_{n}\right)$ condition is verified;
X=0, if the $X\left(d\left(x_{n}\right)\neq j_{n}\right)$ is not verified;
and d(x) represents the classification model, as depicted in Figure 6.

**Figure 7:**
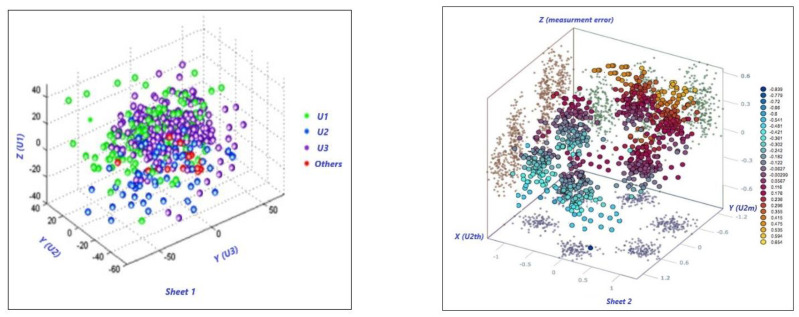
Processing data of Table 2 as measured velocities/flow. The plots display the clustering process related to electrical and flow quantities. Sheet 1 (**left**) depicts a major clustering versus sheet 2 (**right**). Major clustering indicates a less turbulent flow. The conditions reported on the right indicate major turbulence.

The resubstituting estimator is calculated using the same dataset that is used for the construction of the classification model *d*. The above data, along with the previous algorithm have yielded to the results plotted in Figure 7. This latter describes a cluster of direct measurements (sheet 1), and a cluster with more sparsity including measurement error (sheet 2). Neither cluster degenerates into a sphere, both show more or less sparsity. Sheet 1 displays less sparsity because the uncertainty propagation is limited by the instrumentation used to perform the measurement, while Sheet 2 illustrates major sparsity due to the calculation of measurement error that introduces a further uncertainty due to indirect measurements needing propagation connected to the computation. 

In any case, a perfect sphere or quadric cannot be obtained because of the turbulence of the flow, which means that all 3D measurements do not belong to the same spatial positions (or spindles). That is, a fortiori, a confirmation of the conventional approach illustrated in this paper where turbulences have been reported. It is also necessary to validate the clusters using an appropriate indicator. The connection between accuracy and loss could help accordingly. The accuracy is generally defined in artificial intelligence and in machine learning, as reported in Equation (9).

(9) $$\mathrm{Accuracy}=\frac{\mathrm{number}\;\mathrm{of}\;\mathrm{correct}\;\mathrm{predictions}}{\mathrm{total}\;\mathrm{number}\;\mathrm{of}\;\mathrm{predictions}}=\frac{\mathrm{TP}+\mathrm{TN}}{\mathrm{TP}+\mathrm{TN}+\mathrm{FP}+\mathrm{FN}}$$

in which TP stands for true positive, TN is true negative, FP is false positive, and FN denotes false negative.

**Figure 8:**
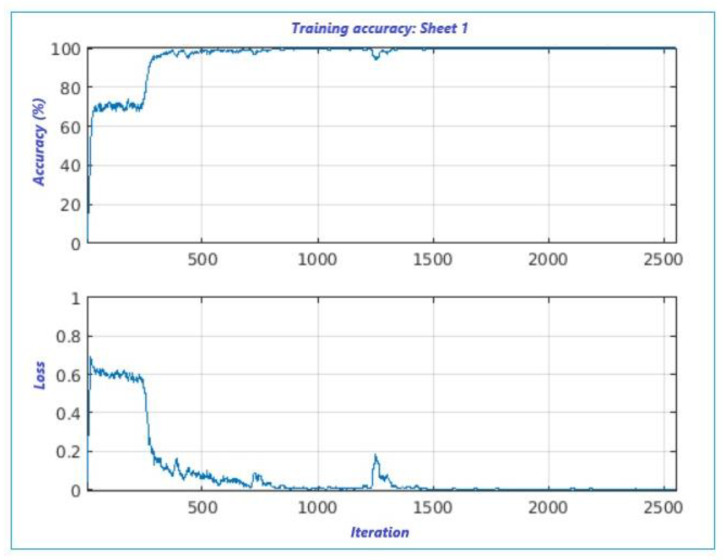
Proposed supervised leaning classification algorithm for sheet 1.

**Figure 9:**
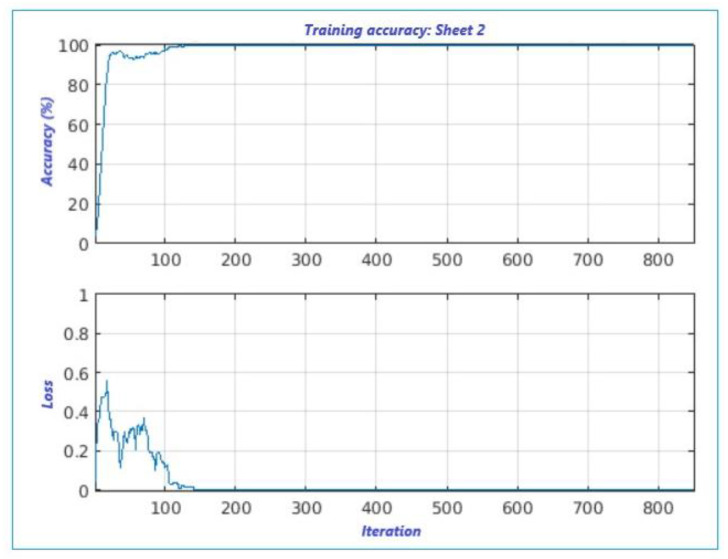
Proposed supervised leaning classification algorithm for sheet 2.

Instead, the loss is an indicator for characterizing the “loss in classification” and regression loss. In this paper, we work on the classification loss [19] to better predict the class of output. Figure 8 shows both accuracy and loss trends versus the number of iterations; analogously, both parameters are illustrated in Figure 9 for the second set of data.

In the first plot, shown in Figure 8, the accuracy is attained after around 400 iterations, with a step-rise around 250 iterations (75%), and a loss around 300 iterations with a loss at 0.6. While the second plot, Figure 9, displays high accuracy at the first step-rise versus the first. It also exhibits a low loss of classification, certainly due to the use of calculated measurement error. We can state that both results shown in Figure 7 are accurate.

## 7. Expanded Metrics for Comparison: Geostatistics and Sobol-Based Sensitivity

Finding the third component is an issue that regards the “missing data” topic, which is a controversial theme. The geostatistics can come to help, given the spatial dimension of the component to be retrieved, by taking into account a specific uncertainty. As is mentioned in the abstract and summary, we need to find the spatial distribution in terms of prediction, that is, the Best Linear Unbiased Estimator called as the Ordinary Kriging [20]. The latter exhibits, as an estimator, the following features:

- it calculates the variance necessary for accuracy;
- it is denoted as an exact estimator because it delivers, in the points where we have the space information, its true value;
- it is based on a model of probabilities by considering error and deviation.

The Kriging estimator [21] is an anticipatory method that is different from the other one because it produces a set of estimates with minimum error variance. The error variance, *σ*^2^*_R_*, of a set of *k* estimates can be written as:

(10) $$\sigma^{2}{}_{R}=\frac{1}{k}\sum_{i=1}^{k}[{\hat{v}}_{i}-v_{i}-\frac{1}{k}\sum_{i=1}^{k}({\hat{v}}_{i}-v_{i}){]}^{2}$$

where $v_{i}$ are the real values and ${\hat{v}}_{i}$ the esteemed values. Assuming an average error equal to zero, the previous equation is set as:

(11) $$\sigma^{2}{}_{R}=\frac{1}{k}\sum_{i=1}^{k}(r_{i}-0{)}^{2}=\frac{1}{k}\sum_{i=1}^{k}[{\hat{v}}_{i}-v_{i}{]}^{2}$$

To minimize the variance of the error, it is necessary to introduce other parameters by adding the Lagrange parameter. By computing the partial derivative and placing it equal to zero, we obtain the set of weights that minimizes the error variance of a quantity through the Kriging method, which depends, however, on the covariance model [22].

(12) $${\tilde{\sigma }}^{2}{}_{R}={\tilde{\sigma }}^{2}-W\cdot D$$

**Figure 10:**
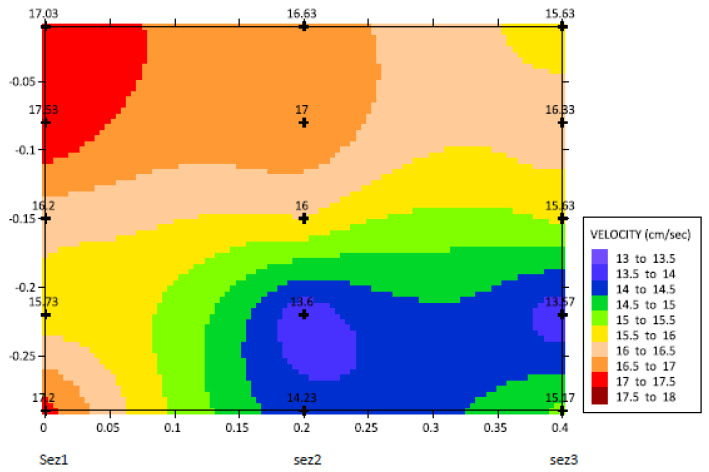
Distribution of predicted velocities on the three sections of the channel: at the beginning (sez1), the middle (sez2), and the end (sez3).

Under the practical point of view, the model chosen for the random function is bound to be the space continuity of the sampled data set. The above representation has been applied to flow measurement [23,24], particularly to detect the third component of Table 2. We can say that the approach adopted in this paper is correct, given the fact that the velocities represented in Figure 10 (+symbols) fall within the range of uncertainty designated in the paper.

**Figure 11:**
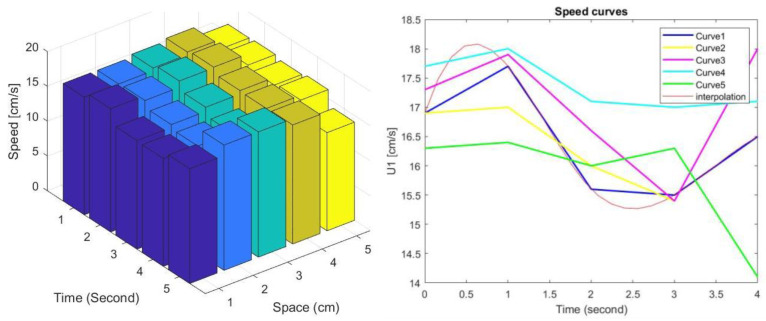
Distribution speed variations (**left**), and sensitivity analysis for *U*_1_ acquisitions of Table 2 (**right**).

The second indicator of this section is the Sobol-sensitivity analysis [25], which is also another way to express the results. This can be accomplished with aid of the Sobol’ indices, which are considered as a variance-based statistical technique for global sensitivity analysis: it allows one to measure the individual importance of each parameter, as well as their joint effect on the model output. Since the 3D flow measurement is complex, the sensitivity analysis permits one to recover the most sensitive and significant parameters encompassed in the measured data. Sobol’s method points out the interactions between the different velocities included in Table 2. Each velocity displays five measurements; let us consider, for instance, *U*_1_ with five measurements at different conditions. Figure 11 indicates all velocities reported on the left side according to the Sobol’s global description, and on the right, we can see the example for one case. The global interpolation encompasses all five curves related to the considered velocity. The sensitivity here demonstrated is close to the Chauvenet’s criterion. Hence, all measurement values are globally coherent, and they are from the same instrument. 

## 8. Discussion and Conclusions

In this work, we have validated a hybrid procedure, which is experimental and computational and apt to reconstruct a 3D incompressible flow field starting from only 2D measurements of the flow coupled to the numerical solution of the continuity equation, which is also useful in industrial applications to reduce the instrumentation cost weight on the global experimentation costs. The validation of the procedure has been based on the evaluation of the uncertainty of the computed quantity, in this case, the stream-wise velocity component as a function of the other two velocity components, measured in the transversal planes, of a forced flow of water in the wake of a NACA 0012 airfoil immersed in a straight rectangular channel.

To this aim, 3D and accurate experimental equipment was utilized, consisting of a 3D Vectrino ADV, available in the applied hydraulics laboratory at the Politecnico di Bari-University, and a high precision 3D system of graduation scales to determine the locations of the measurement points. The stream-wise velocity component measured by the instrument was used as reference quantity, which compared the corresponding computed quantity to evaluate the order of magnitude of the errors derived from the hybrid procedure.

While the accuracy of the numerical model used for the discretization of the continuity equation was limited, here, only a first order scheme was applied for the simplicity of treatment. The obtained results have proven the goodness and efficiency of the technique, which can eventually be improved by the use of higher order numerical discretization schemes, in the event of complex hydraulic configurations dealing with high turbulences.

The numerical prediction of the whole velocity field based on partial experimental data has been suitable for describing the main coherent structures populating the flow behind the airfoil. To discuss the agreement between the theoretical prediction and the physics governing the vortices generation in the wake, we estimated their main spatial and temporal scales. We refer to the Kolmogorov theory of the isotropic and homogeneous turbulence that can be applied in the core region of the channel in which the boundary effects can be neglected.

The Kolmogorov theory makes an energy budget among the large, inertial, and dissipative scales. The large and inertial scales are dominated by the transport of the mechanical energy, epsilon, which is dissipated at the smallest scale because of its viscosity.

The large scales are defined by the mainstream velocity U, being the length scale L in the proposed set-up of the transversal section width. Similarly, the dissipative smallest vortices are characterized by the length scale r, the time scale $T_{r}$, and the velocity scale $U_{r}$, according to the following equation:

(13) $$\epsilon =\frac{U^{3}}{L};$$

(14) $$r={\left(\frac{\nu^{3}}{\epsilon }\right)}^{\frac{1}{4}};$$

(15) $$T_{r}={\left(\frac{\nu }{\epsilon }\right)}^{\frac{1}{2}};$$

(16) $$U_{r}={\left(\nu *\epsilon \right)}^{\frac{1}{4}}.$$

The previous dynamics coexist with the vortices induced by the airfoil, in which a similar analysis can be carried out by using the length scales of the airfoil, NACA 0012, and the chord itself, which is 0.05 m long and the angle attack of 20°; if we refer to the mean undisturbed flow velocity U=0.16 m/s (corresponding to the flow rate running in the channel) and assume the kinematic viscosity $\nu =0.892\times 10^{-6}{\;m}^{2}/s$ for the water, the following values can be estimated for these parameters:

$$\epsilon =8.2\times 10^{-2}\;\frac{m^{2}}{s^{3}};\;r=5.4\times 10^{-5\;}\;m;\;T_{r}=3.3\times 10^{-3}\;s;\;U_{r}=1.6\times 10^{-2}\;m/s\;.$$

These estimations are far from the real topology and dynamics of the coherent structures; nevertheless, they depict the limits that the theoretical model proposed in this paper can be applied in. We can see that the sampling time for the data acquisition is of the same order of the smallest time scale of turbulence *T_r_*. For this reason, the dynamical system is fully predicted in time over all the vertical scales. The spatial distance between the measurement locations is significantly larger than the smallest vortex dimension developed into the flow. Thus, only large scales are resolved in spaces, whereas the smaller ones are filtered by the sampling procedure. This point can be useful for the industrial purposes in design since only the spatial average velocity is taken into account and the velocity fluctuations are neglected. The machine learning-based metrics have added a further contribution in terms of data clustering and accuracy. It could be another opportunity for a new paper on the use of machine learning for this kind of measurement. Additional metrics such as the Kriging estimator and Sobol sensitivity have been used to strengthen the work results.

As indicated in the paper and the aforementioned general considerations, the cost and the maintenance difficulties of a 3D sensor/transducer, to be located in an open channel or a pipe, is not always sustainable, and we need to find an alternate way to obtain the same results with less uncertainty. This method is carried out in this paper. In general, it is possible to reconstruct the 3D representation and thus the missing axis by means of a normal sensor, mostly pressure sensors and video-sensors. In [26], for instance, the authors illustrate how to retrieve the spatial data by means of bi-spectra using a pressure sensor for leak detection in the pipeline. 

We know that experiment as well as confutations are affected by errors, influencing the main structures of the flow. of the main structures in the flow. It is understood that the probable accuracy of such flow parameters is easily assessed, propagating the uncertainties of the independent quantities of which they are a function. The estimate obtained is more than sufficient for practical purposes. The statements of this paper can be used to easily design several devices for industrial aims.

## Figures and Tables

**Table 1:** Vectrino ADV technical specifications [14] used in the experiment.

**Water Velocity Measurements**	
**Maximum profiling range**	0.05 m, 0.01 m (field probe)
**Distance from probe**	6 mm
**Sampling volume diameter**	3 ÷ 15 mm
**Velocity range**	±0.03, 0.1, 0.3, 1, 2.5, 4 m/s (software selectable)
**Accuracy**	±0.5% of measured value ±1 mm/s
**Sampling rate**	1 ÷ 200 Hz
**Echo Intensity**	
**Acoustic frequency**	10 MHz
**Resolution**	Linear scale
**Dynamic range**	25 dB

**Table 2:** Measured velocities.

**U1 (cm/s)**					**V1 (cm/s)**					**W1 (cm/s)**				
**1**	**2**	**3**	**4**	**5**	**1**	**2**	**3**	**4**	**5**	**1**	**2**	**3**	**4**	**5**
16.9	16.9	17.3	17.7	16.3	5.10 × 10^−1^	5.80 × 10^−1^	1.70 × 10^−1^	2.80 × 10^−1^	4.60 × 10^−1^	−1.82 × 10^−1^	−3.20 × 10^−1^	1.90 × 10^−1^	−5.00 × 10^−1^	9.10 × 10^−1^
17.7	17	17.9	18.0	16.4	3.00 × 10^−1^	3.90 × 10^−1^	3.00 × 10^−1^	5.00 × 10^−1^	3.00 × 10^−1^	1.30 × 10	2.40 × 10^−1^	1.50 × 10^−1^	−1.90 × 10^−1^	1.40 × 10^−1^
15.6	16	16.6	17.1	16.0	6.80 × 10^−1^	2.10 × 10^−1^	3.80 × 10^−1^	9.10 × 10^−1^	2.70 × 10^−1^	1.10 × 10	4.70 × 10^−1^	−4.00 × 10^−1^	3.70 × 10^−1^	1.70 × 10^−1^
15.5	15.4	15.4	17.0	16.3	2.00 × 10^−1^	1.20 × 10^−1^	4.20 × 10^−1^	3.10 × 10^−1^	0.120	−5.30 × 10^−1^	−3.60 × 10^−1^	4.00 × 10^−1^	4.30 × 10^−1^	3.80 × 10^−1^
16.5	18	18	17.1	14.1	1.00 × 10^−1^	4.30 × 10^−1^	4.30 × 10^−1^	6.80 × 10^−1^	7.00 × 10^−2^	3.50 × 10^−1^	6.20 × 10^−1^	1.40 × 10^−1^	3.70 × 10^−1^	1.00 × 10^−1^
**U2 (cm/s)**					**V2 (cm/s)**					**W2 (cm/s)**				
**1**	**2**	**3**	**4**	**5**	**1**	**2**	**3**	**4**	**5**	**1**	**2**	**3**	**4**	**5**
16.8	16.6	17.4	15.9	16.5	−1.80 × 10^−2^	−3.10 × 10^−2^	2.00 × 10^−2^	−4.90 × 10^−2^	9.00 × 10^−2^	2.70 × 10^−1^	−1.30 × 10^−1^	1.30 × 10^−1^	5.50 × 10^−1^	3.20 × 10^−2^
16.4	16.3	17.0	17.6	17.7	1.00 × 10^−1^	2.40 × 10^−2^	1.50 × 10^−2^	−1.90 × 10^−2^	1.40 × 10^−2^	8.00 × 10^−3^	1.90 × 10^−1^	−2.40 × 10^−1^	1.80 × 10^−1^	−8.70 × 10^−2^
16	16.6	16.5	15.5	14.9	1.20 × 10^−1^	4.70 × 10^−2^	−4.80 × 10^−2^	3.30 × 10^−2^	1.70 × 10^−2^	6.20 × 10^−2^	−2.70 × 10^−1^	2.50 × 10^−1^	3.30 × 10^−1^	2.30 × 10^−1^
13.3	13.3	13.6	13.9	14.1	−5.40 × 10^−2^	−3.40 × 10^−2^	3.80 × 10^−2^	4.30 × 10^−2^	3.80 × 10^−2^	9.60 × 10^−2^	−8.80 × 10^−1^	−2.10 × 10^−1^	−2.60 × 10^−1^	1.20 × 10
14.4	13.6	14.7	13.5	14.7	3.50 × 10^−2^	6.30 × 10^−2^	1.40 × 10^−2^	3.70 × 10^−2^	1.00 × 10^−1^	5.50 × 10^−1^	6.60 × 10^−1^	−2.10 × 10^−1^	2.00 × 10	9.20 × 10^−3^
**U3 (cm/s)**					**V3 (cm/s)**					**W3 (cm/s)**				
**1**	**2**	**3**	**4**	**5**	**1**	**2**	**3**	**4**	**5**	**1**	**2**	**3**	**4**	**5**
14.1	14.9	16.1	16.6	15.9	−2.90 × 10^−3^	7.80 × 10^−3^	2.50 × 10^−3^	2.80 × 10^−3^	−4.40 × 10^−3^	3.00 × 10^−2^	−1.80 × 10^−2^	2.50 × 10^−2^	1.80 × 10^−2^	3.40 × 10^−2^
16.2	16.5	16.3	16.1	16.6	2.50 × 10^−3^	2.50 × 10^−3^	3.30 × 10^−3^	1.60 × 10^−3^	6.70 × 10^−3^	2.50 × 10^−2^	2.50 × 10^−2^	3.30 × 10^−2^	1.60 × 10^−2^	6.70 × 10^−2^
14.1	16.0	17.6	15.7	15.2	7.00 × 10^−3^	8.60 × 10^−3^	−4.70 × 10^−3^	6.50 × 10^−3^	−3.00 × 10^−4^	5.00 × 10^−2^	8.60 × 10^−2^	−4.30 × 10^−2^	−2.50 × 10^−2^	−1.10 × 10^−3^
13.6	13.2	14.4	13.9	13.0	2.70 × 10^−3^	4.40 × 10^−3^	6.40 × 10^−3^	1.80 × 10^−3^	9.90 × 10^−3^	3.70 × 10^−2^	6.30 × 10^−2^	5.40 × 10^−2^	1.80 × 10^−2^	1.00 × 10^−3^
14.8	13.1	15.3	15.4	15.5	7.80 × 10^−3^	8.10 × 10^−3^	1.10 × 10^−2^	6.30 × 10^−3^	2.20 × 10^−3^	6.60 × 10^−2^	7.00 × 10^−2^	1.30 × 10^−1^	4.30 × 10^−2^	1.20 × 10^−2^

**Table 3:** Velocity component *u*_2*−calculated*_ from the continuity equation.

*u*_2−*calculated*_ (cm/s)					
	1	2	3	4	5
1	16.6	16.7	17.3	17.5	16.5
2	17.4	17.6	18.1	18.5	16.7
3	15.9	16.6	16.0	17.1	16.8
4	14.9	15.4	15.7	15.9	16.0
5	16.2	17.1	18.9	16.9	13.3

**Table 4:** Computed % errors between $u_{2_{i,j}-calc}$ and $u_{2_{i,j}-meas}$.

	1	2	3	4	5
1	−0.01	0.01	−0.01	0.10	0
2	0.06	0.08	0.06	0.05	−0.06
3	−0.01	0	−0.03	0.10	0.13
4	0.12	0.16	0.15	0.14	0.13
5	0.13	0.26	0.29	0.25	−0.10

**Table 5:** Mean square errors of the measured *u* at the inflow plane.

$S_{u_{1}}$ (cm/s)					
	1	2	3	4	5
1	0.020	0.021	0.021	0.021	0.021
2	0.022	0.020	0.021	0.019	0.019
3	0.030	0.022	0.022	0.022	0.024
4	0.029	0.034	0.037	0.032	0.040
5	0.024	0.017	0.019	0.023	0.030

**Table 6:** Computed $u_{j,k}$ component in the middle plane and its conservative uncertainty (rounded to the 2nd decimal digit).

	**1**	**2**	**3**	**4**	**5**	**±**		**1**	**2**	**3**	**4**	**5**	cm/s
1	16.6	16.7	17.3	17.5	16.5		1	0.19	0.19	0.19	0.19	0.19	
2	17.4	17.6	18.1	18.5	16.7		2	0.19	0.19	0.19	0.20	0.19	
3	15.9	16.6	16.0	17.1	16.8		3	0.19	0.19	0.19	0.19	0.19	
4	14.9	15.4	15.7	15.9	16.0		4	0.18	0.19	0.19	0.19	0.20	
5	16.2	17.1	18.9	16.9	13.3		5	0.19	0.19	0.20	0.19	0.18	

## Data Availability

Not applicable, own experimental system.
